# Impact of decision-making system in social navigation

**DOI:** 10.1007/s11042-021-11454-2

**Published:** 2022-01-13

**Authors:** Jonatan Ginés Clavero, Francisco Martín Rico, Francisco J. Rodríguez-Lera, José Miguel Guerrero Hernandéz, Vicente Matellán Olivera

**Affiliations:** 1 Escuela Internacional de Doctorado, Rey Juan Carlos University, Móstoles, Spain; 2 Intelligent Robotics Lab, Rey Juan Carlos University, Fuenlabrada, Spain; 3 Escuela de Ingenierías Industrial e Informática, Universidad de León, León, Spain; 4 Supercomputación Castilla y León, SCAYLE, León, Spain

**Keywords:** Social robot, Social navigation, Proxemic, Human-aware navigation, Cognitive architecture

## Abstract

Facing human activity-aware navigation with a cognitive architecture raises several difficulties integrating the components and orchestrating behaviors and skills to perform social tasks. In a real-world scenario, the navigation system should not only consider individuals like obstacles. It is necessary to offer particular and dynamic people representation to enhance the HRI experience. The robot’s behaviors must be modified by humans, directly or indirectly. In this paper, we integrate our human representation framework in a cognitive architecture to allow that people who interact with the robot could modify its behavior, not only with the interaction but also with their culture or the social context. The human representation framework represents and distributes the proxemic zones’ information in a standard way, through a cost map. We have evaluated the influence of the decision-making system in human-aware navigation and how a local planner may be decisive in this navigation. The material developed during this research can be found in a public repository (https://github.com/IntelligentRoboticsLabs/social_navigation2_WAF) and instructions to facilitate the reproducibility of the results.

## Introduction

In recent years, robots have come out from factories and research laboratories and are beginning to populate human-populated domestic and work environments. For years, researchers have identified many challenges related to this presence and its perception by humans. In particular, we are interested in social navigation. This term’s social component refers to the fact that this task has to consider people and the social conventions humans apply when moving between and towards other humans.

The *proxemic* is the discipline that studies how we manage spaces in our social and personal interactions with other individuals. In robotics, many Human-Robot Interaction works use this concept to create more natural and comfortable human interactions. Our research applies the principles of proxemic in navigation. Our proposal goes beyond calculating the distance between the robot and the people. We also assess the robot’s position to a person when interacting with it, even while moving. We want a robot to guide, follow or accompany a person alongside while moving. This work is encompassed within a research project where the authors have been working on the following research problem: *A social robot aiming to interact with humans needs to adapt its navigation behavior when deployed in a real-world environment*.

However, so far, the authors have been solving four research questions (RQ):

- **RQ 1**: How are the areas of human-robot interaction modified at runtime?
- **RQ 2**: What factors change people’s proxemic zones when interacting with a robot?
- **RQ 3**: What are the effects of different behavioral decision-making systems in the activity-aware navigation system?
- **RQ 4**: What are the effects of the different local planners in the activity-aware navigation system?

Previous work explored an answer to question 2 [21], working with people’s moods to adapt to the individual proxemic zones. In that study, the robot adapts the navigation system avoiding personal distances when interacting with humans in a bad mood.

Besides, the study presented in [10] proposed a framework for adapting individuals’ proxemic shape and added a new layer called the cooperation zone, answering Research Question 1. That work measured the time spent in different proxemic zones during the human-robot interaction.

This paper is focused on questions three and four. It is examined and evaluated the effects of the decision-making system over the human-aware navigation component. Besides, it is explored the influence of different local planners in human-aware navigation performance. Thus, the contribution of this paper is an explanatory and evaluation approach for analyzing the cognitive architecture’s impact and measuring the performance. We will describe the framework’s integration using two different approaches to decision-making systems: Behavior Trees [13] and AI Planning [24]. Behavior Trees are a reactive approach to robot control. AI Planning is a classic and deliberative approach to decision-making in the performance of a robot. Both methods have been evaluated in a simulator with a focus on answering the previously proposed research questions. This simulator presents a realistic situation in which we evaluate the application of our framework in human interaction.

This paper is structured as follows: In Section 2, we will present all the works we consider relevant in human-aware navigation. Section 3 will briefly describe the framework, how the proxemic zones are built, and the different approaches to implement a decision-making system for a cognitive architecture. Section 4 shows experiments that validate our approach, and Section 5 contains its results. Section 6 contains a brief discussion about the experiment results, and finally, Section 7 contains the present work conclusions.

## Background

The problem of adapting the navigation system for considering the individuals’ needs and their social conventions is well known in the robotics field. One of the most extended social navigation models is the use of proxemic theory [23]. The proxemic zone defines the space around a person. It is based on four different areas: intimate, personal, social, and public. In this work, we will focus on the intimate and personal areas.

- **The intimate zone** (< 0.4 m around the person): Navigation is forbidden in this zone to avoid collisions and does not disturb humans.
- **The personal zone** (0.4 m–1.2 m around the person): In this zone, the person interacts with known people or performs a collaborative task. Some works [3, 26, 41] consider this zone as a restricted zone for robot navigation. We propose that this zone be adaptive because, depending on the context, it will be a restricted or a cooperation zone where the robot enters to carry out a task with the person.

The proposed system can adjust to situations where robots and human beings close or scenarios such as the current pandemic caused by COVID-19, where the safety distance of 2 m must be respected. The proxemic theory defines areas that could change according to the context, culture, or age. Following this idea, in [42], the authors propose that the proxemic zones are dynamic and are modified with human intention. Other works have developed methods to follow the social convention of keep on the right when walking in a corridor using this theory as a base [30, 36].

The proxemic theory is a great solution to model people in the space but needs the rest of robot capabilities to be useful, i.e., navigation, dialogue, or perception. Researchers face an old, recurrent, and still open problem in artificial intelligence-based systems and social robotics when organizing the autonomous system capabilities: how to organize perception and actuation in an *intelligent* way. There are three types of architectures to organize the robot’s behaviors: behavior-based, symbolic, and hybrid. These types refer to the “cognitive” dimension, each of them having different origins. Therefore, behavior-based architectures have a biological inspiration, symbolic approaches are more related to psychology, and hybrid ones could be considered engineer-inspired. Kotseruba et al. show in [28] a complete analysis of different and more detailed ways of classifying architectures.

The present work uses a hybrid architecture to organize the capabilities, i.e., an architecture with deliberative and behavior-based layers. There are multiple ways to materialize the deliberative layer, for example, Fuzzy logic, Policy-based approaches, FSM (Finite State Machines), Petri nets [14], Behavior Trees, or IA Planning, among other forms. Fuzzy logic or Policies based approaches are more oriented to control or reactive decision-making instead of orchestrating a set of behaviors, although there are works where fuzzy [38] or MPDM (Multi-Policy Decision Making) is used as deliberative layer [15]. The FSM algorithm provided satisfactory results in many mobile robot research works [17], but with growing numbers of states and transitions, modeling complex behaviors with FSMs can become too complicated for real-world tasks. Petri nets have a larger modeling power than FSM and can model the state space with a smaller graph. It is composed by modules that can be modeled separately and then composed with others. Because of this, Petri nets usually lead to exponential growth in the state space. In some cases, the generation of all states with Petri nets leads to the construction of a large network, complicating the network analysis.

In this paper, the evaluation of Behavior Trees and IA planning is proposed as a decision-making system for a social robot navigation [7]. Behavior trees (BTs) are successfully for task planning in robotics as a replacement for finite state machines (FSM) [2]. The use of BTs simplifies the decision-making process [1]. Behavior trees use a structured traversal approach to replace the growing mess of state transitions of FSMs. Complex states are easy to define in BTs, and it is very easy to see the logic in BTs. They are fast to execute and easy to maintain. BTs have also been applied successfully to execute robot manipulation and complex mobile-manipulator tasks [13].

Planning Definition Domain Language (PDDL) [20] was a significant step for the standardization of planning languages. This language, inspired by STRIPS, has become a standard that has driven this area of research. One of their releases, PDDL 2.1 [18], was especially relevant as it included multiple enhancements, such as the ability to use temporal planning with durative actions, which introduces the concept of time into the PDDL framework. Planning and Robotics are converging again through various planning systems and frameworks for generating behaviors for robots in recent years. ROSPlan [9] is one of the most successful current planning frameworks and is a reference in ROS [37]. PlanSys2^1^ is its evolution to the new ROS generation (ROS2) with a lot of new features.

This work focuses on IA Planning and BTs as a decision-making approach because it is emerging expansion as mission controller systems in mobile and social robotics to solve complex tasks. Thanks to the frameworks described before, we can easily and quickly develop different behaviors using these technologies.

## Framework description

**Fig. 1:**
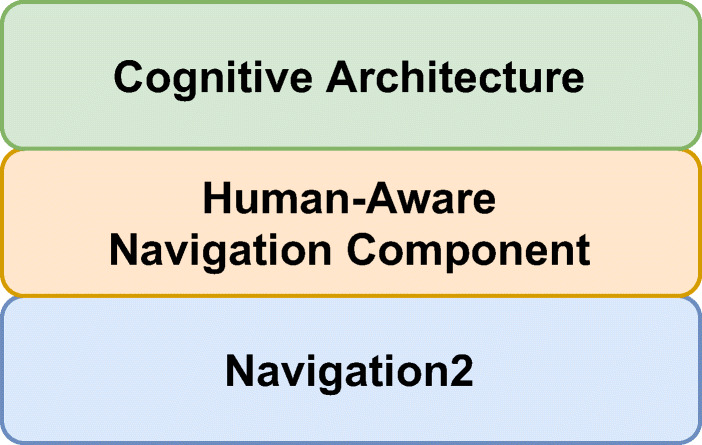
Layered system with the components involved in our approach

This section illustrates the different components involved in this study (Fig. 1. From a bottom-up approach, the main components involved are:

- Navigation 2
- Human-Aware Navigation Component
  - Human-Aware Framework supporting proxemic and cooperation zones
- Cognitive Architecture
  - Behavioral Decision-Making System

### Navigation 2

Nav2 [32] is the new ROS2 navigation system, an evolution from ROS Navigation Stack. This system seeks to find a safe way to have a mobile robot move from point A to B. It will complete dynamic path planning, compute velocities for motors, avoid obstacles, and structure recovery behaviors. The Nav2 system was designed for a high degree of customizability and future expansion. Because of this, we have chosen to integrate the proposed human representation framework as a cost map layer, showing in-depth in [10].

### Human-aware navigation component

This work uses the framework proposed in [10] for representing the space surrounding a person, the proxemic areas, on a cost map. This representation is fundamental to differentiate humans from the rest of the obstacles, thus enriching the robot’s knowledge of its environment.

#### Human-aware framework supporting proxemic and cooperation zones

This article used Asymmetric Gaussian proxemic zones proposed in [25] instead of proxemic zones based on Gaussian functions of concentric circles [21].

**Fig. 2:**
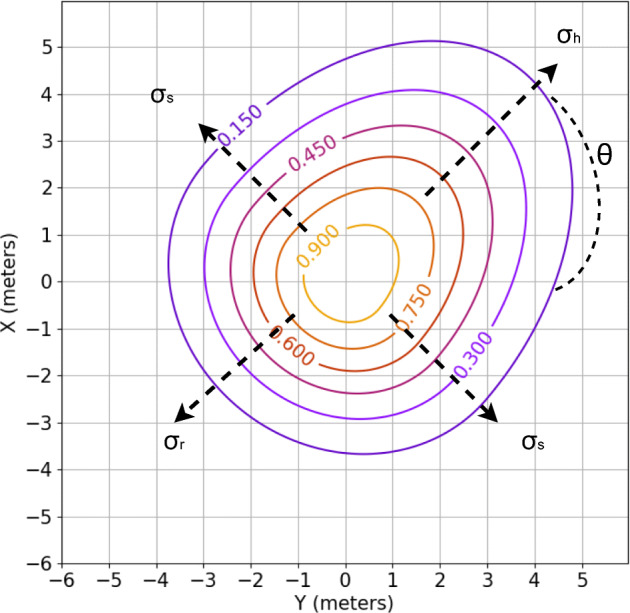
Asymmetric Gaussian centered at (0, 0), rotated by *Θ* = *π*/4, with variances *σ*_*h*_ = 3.0, *σ*_*s*_ = 2.0, and *σ*_*r*_ = 1.8

**Fig. 3:**
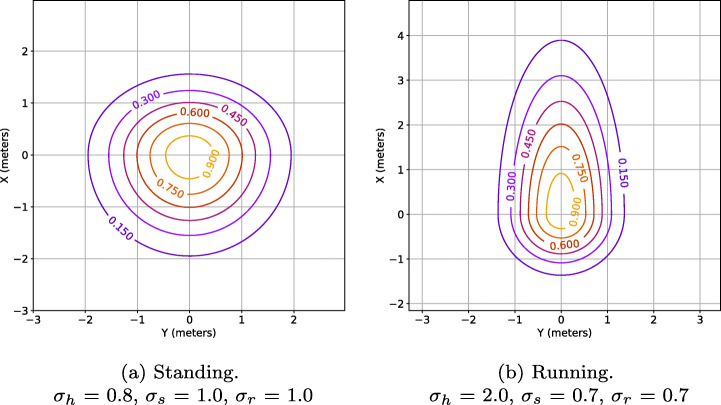
Different proxemic shapes based on the context information

This proxemic provides a high adaptation capacity to the environment because we can modify their size and shape, unlike previous research that only modified their size. The Asymmetric Gaussian are defined by four variables: head (*σ*_*h*_), side (*σ*_*s*_), rear (*σ*_*r*_) and orientation (*Θ*). Figure 2 shows a graphic explanation of these parameters. Thanks to the high adaptability of this proxemic, we can associate different proxemic shapes and sizes with different activities of human’s daily life. Thus, we can build a social map in which people are represented differently based on their activity (Fig. 3).

#### Asymmetric Gaussian function as human activity representation

The proposal to represent people and their activities uses the model described in [25]. In this model, people generate areas where navigation is forbidden or penalized, using an asymmetric Gaussian function. Let P_*n*_ = {p_1_,p_2_...p_*n*_} be the set of n persons detected in the scenario and p_*i*_ = (x, y, *𝜃*) is the pose of the person i.

$$ \begin{array}{@{}rcl@{}} g_{pi}(x,y) &=& e^{-(A(x-x_{i})^{2}+2B(x-x_{i})(y-y_{i})+C(y-y_{i})^{2})} \end{array} $$

With A, B, C:

$$ \begin{array}{@{}rcl@{}} A &=& \frac{\cos(\theta)^{2}}{2\sigma^{2}} + \frac{\sin(\theta)^{2}}{2{\sigma_{s}^{2}}} \end{array} $$

$$ \begin{array}{@{}rcl@{}} B &=& \frac{\sin(2\theta)}{4\sigma^{2}} - \frac{\sin(2\theta)}{4{\sigma_{s}^{2}}} \end{array} $$

$$ \begin{array}{@{}rcl@{}} C &=& \frac{\sin(\theta)^{2}}{2\sigma^{2}} + \frac{\cos(\theta)^{2}}{2{\sigma_{s}^{2}}} \end{array} $$

where *σ* s, as already mentioned, is the variance on the left and right and *σ* = (*α* ≤ 0? *σ* r : *σ* h).

Using this model allows us to create areas around people detected with different sizes and shapes. Figure 3 shows two activities’ representations. If a person is moving with a determined velocity in the robot’s surroundings (Fig. 3b), the proxemic zones will be updated with the human’s velocity estimation, updating the *σ*_*h*_ parameter from the model. Thus, it creates a big zone in a person’s front where navigation is forbidden or penalized, avoiding hit with a person in a hurry and with a dynamic size, based on the velocity estimation.

**Fig. 4:**
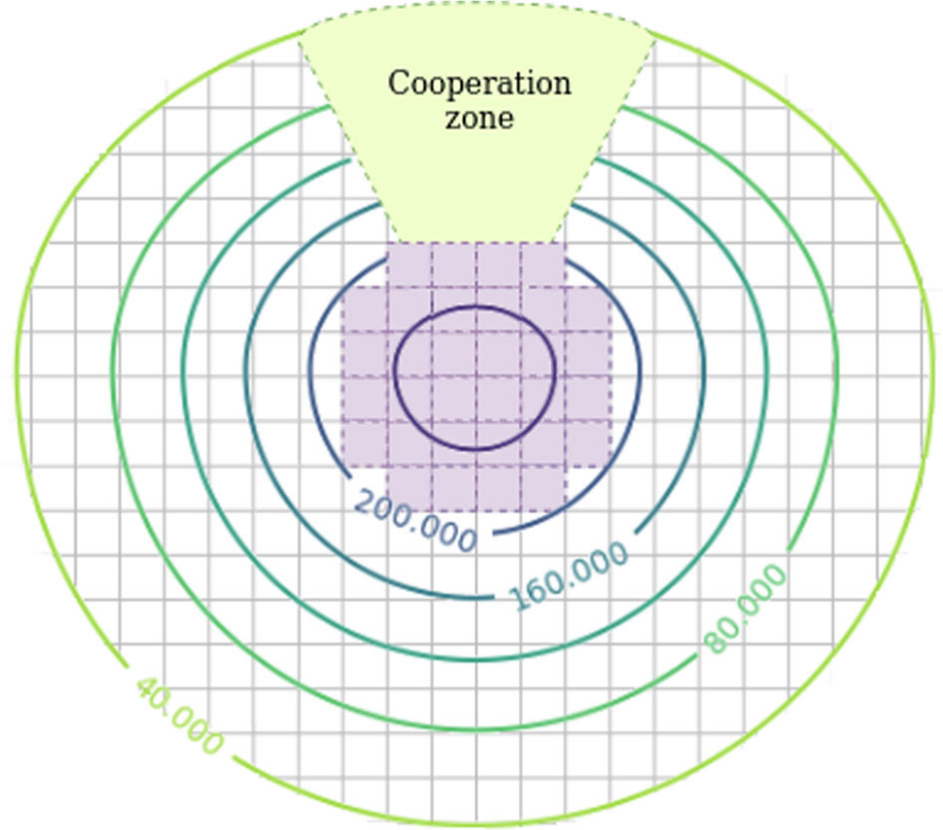
Our novel proxemic zone with a cooperation zone to improve the human-robot activities

Also, we propose new proxemic shapes oriented to improve the performance of collaborative tasks between robots and humans, taking as reference the work of Mead et al. [33]. They show that humans adapt their proxemic zones to interact with a robot. In that way, we have designed proxemic zones that contain a *cooperation zone* (Fig. 4). The robot occupies this zone during a collaborative task to keep close to the person, e.g., a conversation, a robot following a human or robot and, human walking side-to-side. These zones are located within the personal zone but always respecting the intimate zone.

#### Adaptation of the proxemic areas, the cooperation zone

When we want to create areas around people where navigation is forbidden, the use of proxemic zones is the most extent method [29]. On the opposite side, robots have to be social, natural and perform daily tasks with humans [5]. We can see how these two concepts collide, one restricts navigation around people, and the other promotes human-robot collaboration and interaction. To deal with this situation, is necessary to create a representation that takes into account the comfort and safety of people and at the same time allows the execution of social tasks, e.g., approaching a person to give him a voice message or a robot receptionist accompanying a person to a meeting room.

The presented article proposes the creation of cooperation zones, Fig. 4. A cooperation zone is located outside the intimate zone and inside the personal zone, so the robot will keep a reasonable distance to avoid colliding and allowing a comfortable and natural interaction. We code this zone as a free zone on the map to allow fluid navigation inside the cooperation zone. The cooperation zones are configurable and can be set from 0 to 2 zones for each person. They are located in the desired orientation and size, depending on the task for which they are designed. In this way, a cooperation zone to facilitate HRI tasks is located in an orientation equal to the person, or a cooperation zone designed to walk side-to-side with a human is composed of two subzones on the human’s side.

### Decision-making systems

This research proposes how different decision-making systems affect social navigation. Factors such as decision-making reaction time can be crucial for a robot to efficiently react to changes in proxemic zones due to interactions or changes in the robot’s mission. In this work, we use two decision-making methods that we consider representative in this type of system:

- **Behavior Trees**: It is a framework widely used currently to code tasks in a reactive and modular way.
- **AI Planning**: It is a deliberative approach in which a plan is calculated to achieve a goal.

#### Behavior trees

Behavior Trees [13] is a framework to code the execution of actions through a tree. The nodes in this tree are functional elements that can be:

- **Action nodes**: The tree leaves represent actions to be carried out or conditions to check.
- **Control nodes**: The rest of the nodes, those with child nodes, define the execution flow of the tree. They implement different control approaches, the most common are:
  - **Sequence**: When a sequence node is ticked, it ticks its children. Each tick is made to a single child, starting with the one furthest to the left. When it returns SUCCESS, it is passed to the next child. If any of the children returns FAILURE, the sequence node returns FAILURE. The sequence node returns SUCCESS if all children have returned SUCCESS. In any other case, it returns RUNNING.
  - **Condition**: Returns SUCCESS if the evaluation of the conditions results in true, and FAILURE for false.

**Fig. 5:**
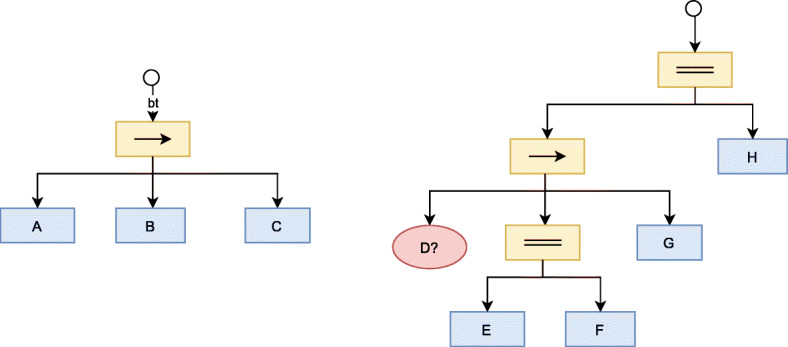
Two different behavior trees. Yellow rectangles are control nodes (→ for sequences and = for parallel execution). Red ellipse is a condition node, and blue rectangles are action nodes

The main operation is *tick*. When a node is ticked, this operation can return SUCCESS if the node function is completed successfully, FAILURE if the node function failed, or RUNNING if the node function has not finished yet. When the root of a Behavior Tree is ticked, it executes until it returns SUCCESS or FAILURE. This tick is propagated through the tree by the *control nodes*. Figure 5 shows several examples of Behavior Trees.

**Fig. 6:**
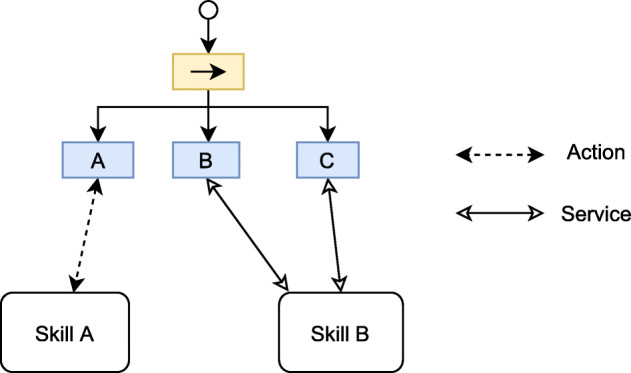
Leaves nodes controlling the execution of external subsystems

In most decision-making systems, the BTs leaves do not usually directly produce control commands to the robot but rather orchestrate other subsystems’ execution. Figure 6 shows how the tree leaves use actions or services to request other subsystems to perform a task. In a robotic system, these subsystems usually correspond to skills, such as navigation, manipulation, dialogue, among others.

#### AI planning

PDDL [18] defines a standard to code symbolic planning problems. Instead of indicating the steps to achieve a goal, planning defines a set of actions with the requirements to be applied and their effects when they are carried out. A plan contains the sequence of actions that should be executed given a goal to be achieved.

In planning there are the following main elements:

- **The domain** (Listing 1) defines the type, predicates and actions available. Here are defined the requirements and effects of every action.
- **The problem** (Listing 2) to solve. Given the types and predicates of a domain, the problem contains the concrete instances and predicates that it starts from. It also contains a specific goal to be solved.
- A plan (Listing 3) as a sequence of grounded actions that must be executed to acquire the goal.

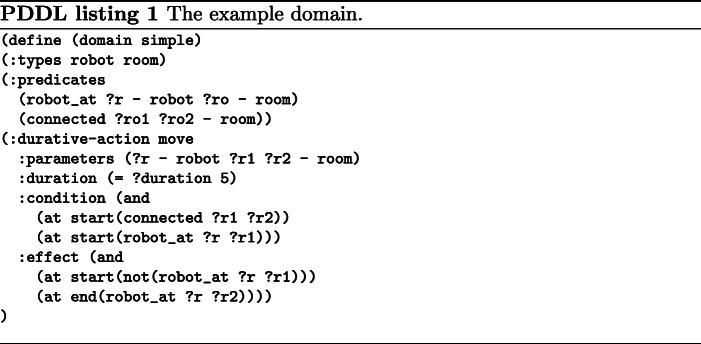

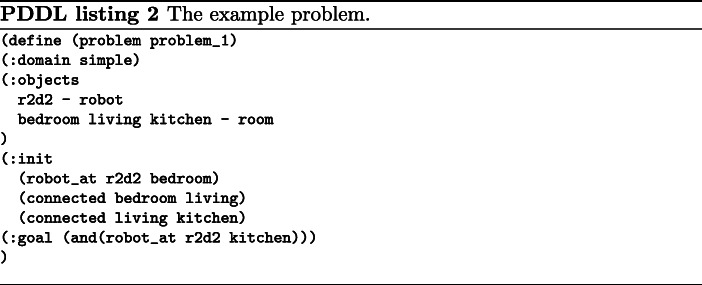

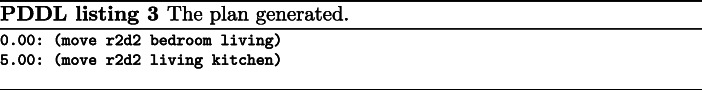


**Fig. 7:**
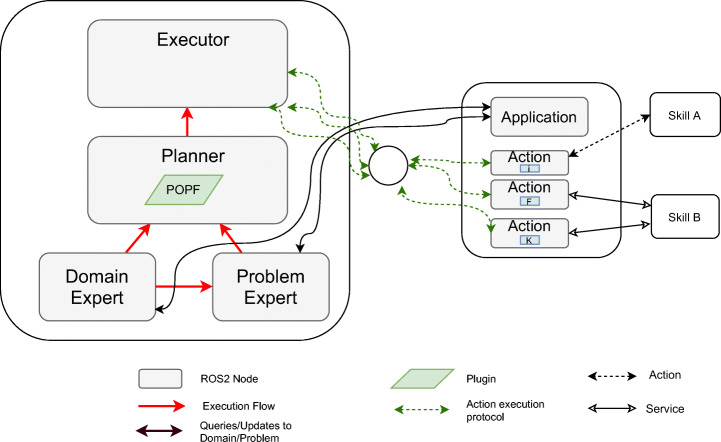
IA Planning working as decision-making system

The decision-making system based on PDDL is implemented through the planning system (Fig. 7). This system has the following components:

- A module that contains the PDDL model, which we will call the *Domain Expert* and a module called *Problem Expert* that loads the problem file generated using the domain and the knowledge base.
- A module that calls a PDDL plan solver (POPF [11] in this case) that we will call *Planner*. It receives the domain and the problem, and it generates the plan. The plan contains the sequence of actions to reach the goal.
- A module that is responsible for executing a plan, which we will call *Executor*. This module reads a plan and dispatches each of the actions to the processes that carry them out. Each application contains a module that implements each of the actions of the model. This module also verifies the requirements at runtime, aborting the plan if they are not met.

### The cognitive architecture

The use of a cognitive architecture allows a robot to perform real, long-term, and high variability tasks thanks to the different tools that typically offer: long-term memory or knowledge representation, orchestration and interaction interfaces.

**Fig. 8:**
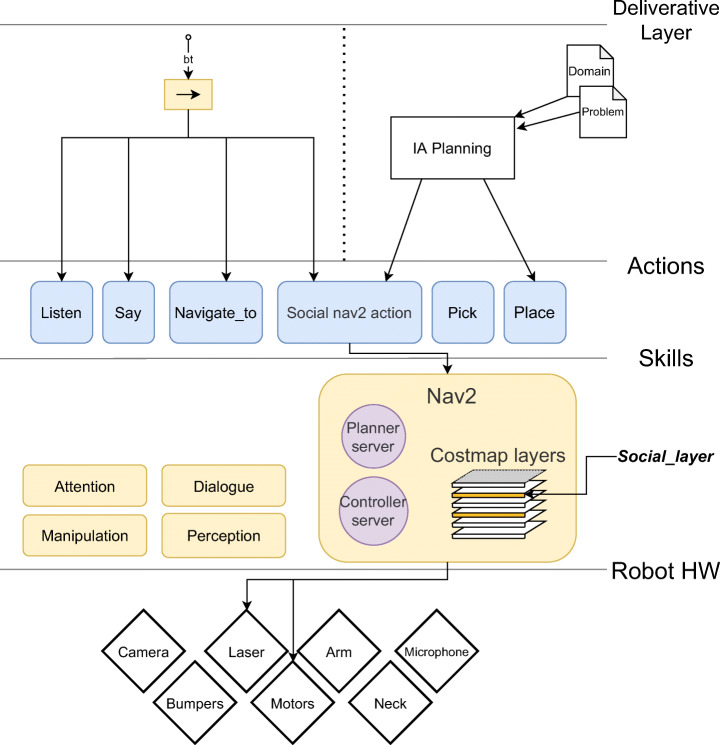
Layered cognitive architecture

Figure 8 shows the cognitive architecture design supported on layers.

- Deliberative layer: this level contains the decision-making that define the modes and behaviors of the robot at a high level. The implementation of this level could be based on PDDL or Behavior trees, as we explained before.
- Actions: contains the implementation of the actions defined in the upper layer. This level is the bridge between both paradigms.
- Skills: The skills can be activated from actions and can be reused from any of the actions. This level includes navigation, visual attention, dialogue, and manipulation modules, among others. Our navigation skill is based on Nav2 system. In this one, the global cost map is used by the planner to calculate the path from the robot’s current position to target, and the local cost map is used by the controller generating movements to follow this path, avoiding unexpected obstacles. Both the global cost map and the local cost map result from combining the different layers [31]. We have created a new layer, the *social_layer*, to integrate our dynamic proxemic representation framework in the navigation system.
- Robot hardware: Hardware components of the robot, including its sensors.

## Experimental setup

**Fig. 9:**
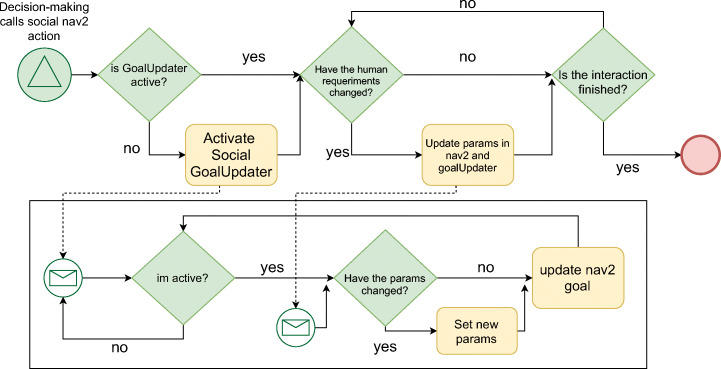
Information flow between the architecture components

The experiments carried out aim to demonstrate the strategies for running the navigation component and the impact on the overall architecture running ROS2. For this purpose, we will compare the two decision-making approaches presented in Section 3.3. Moreover, we will show how the proposed framework is integrated with a dialog module, providing the system the ability to react to human intentions or feelings. Figure 9 shows the information flow between the architecture components.

When the symbolic layer calls the *social nav2* action, it activates the *goalUpdater* module. It calculates the best robot position to do a collaborative task with a human, e.g., the interaction position different from the side-to-side walk position. Once the goalUpdater module is actives, the system checks if the human has changed the interaction requirements. The person could feel uncomfortable and requests the robot to walk away or, conversely, requests the robot to get close if necessary. These requests affect the proxemic zones’ size and shape, i.e., modifies the Asymmetric-Gaussian parameters. GoalUpdater module sets the new parameters in the Nav2 stack, if those have changed, and updates the navigation goal. We get a robot collaborating with humans, adapting to the context and human preferences with this process.

All experiments were performed in a simulation environment, on a computer with an Intel Core i7-10875H 2.3GHz processor with 32 GB of DDR4 RAM, GeForce RTX 2070, and Ubuntu GNU/Linux 20.04 using Gazebo as simulator and ROS2 Foxy as robot framework.

### Software

PlanSys2 [39], which is an IA planning framework, has been used to implement the PDDL approach. It uses POPF [12] as a planner, among others. Using this tool, we can easily decompose complex tasks into a sequence of more uncomplicated actions.

To implement the behavior tree approach, we have used the library BehaviorTree.CPP,^2^ an open-source C++ library supporting type-safe asynchronous actions, composable trees, and logging/profiling infrastructures for development.

There are two main control algorithms in Nav2: TEB (Timed Elastic Bands) developed by Rosmann [40] and DWB,^3^ the updated version of DWA [19] (Dynamic Windows Approach). TEB allows the consideration of the robot’s dynamic constraints and direct modification of trajectories rather than the global path. The “timed elastic band” is formulated as a scalarized multi-objective optimization that depends on a few consecutive configurations rather than the entire trajectory. DWA/DWB is a velocity space-based local reactive avoidance technique where a search for commands controlling the robot is carried out directly in the space of velocities. The trajectory of a robot can be described by a sequence of circular and straight-line arcs.

Another fundamental tool for performing the experiments is the pedestrian simulator based on the social force model, PedSim [4, 22]. This simulator will provide people’s position and orientation in each instant of time.

Finally, we have used TIAGo robot from PAL Robotics as mobile robot platform, and we have used tf_pose_estimation,^4^ a TensorFlow implementation of OpenPose [8], to detect persons and their position. This software replaced PedSim in the real-robot experiments.

### Scenarios

**Fig. 10:**
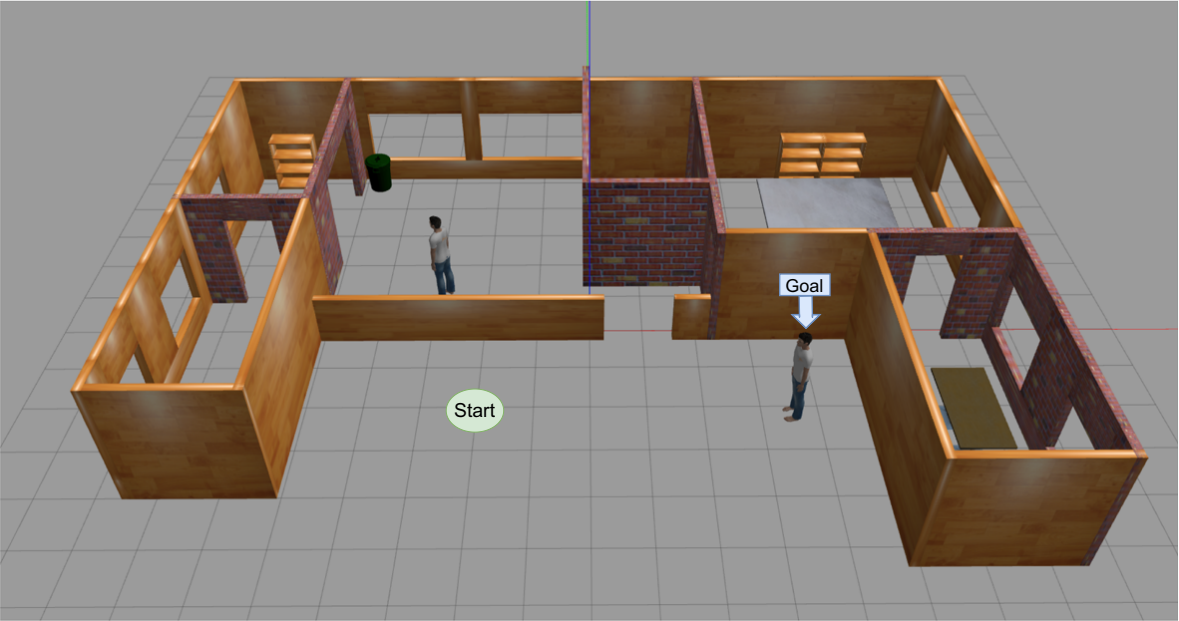
Domestic scenario from Gazebo simulator with experiment waypoints

**Fig. 11:**
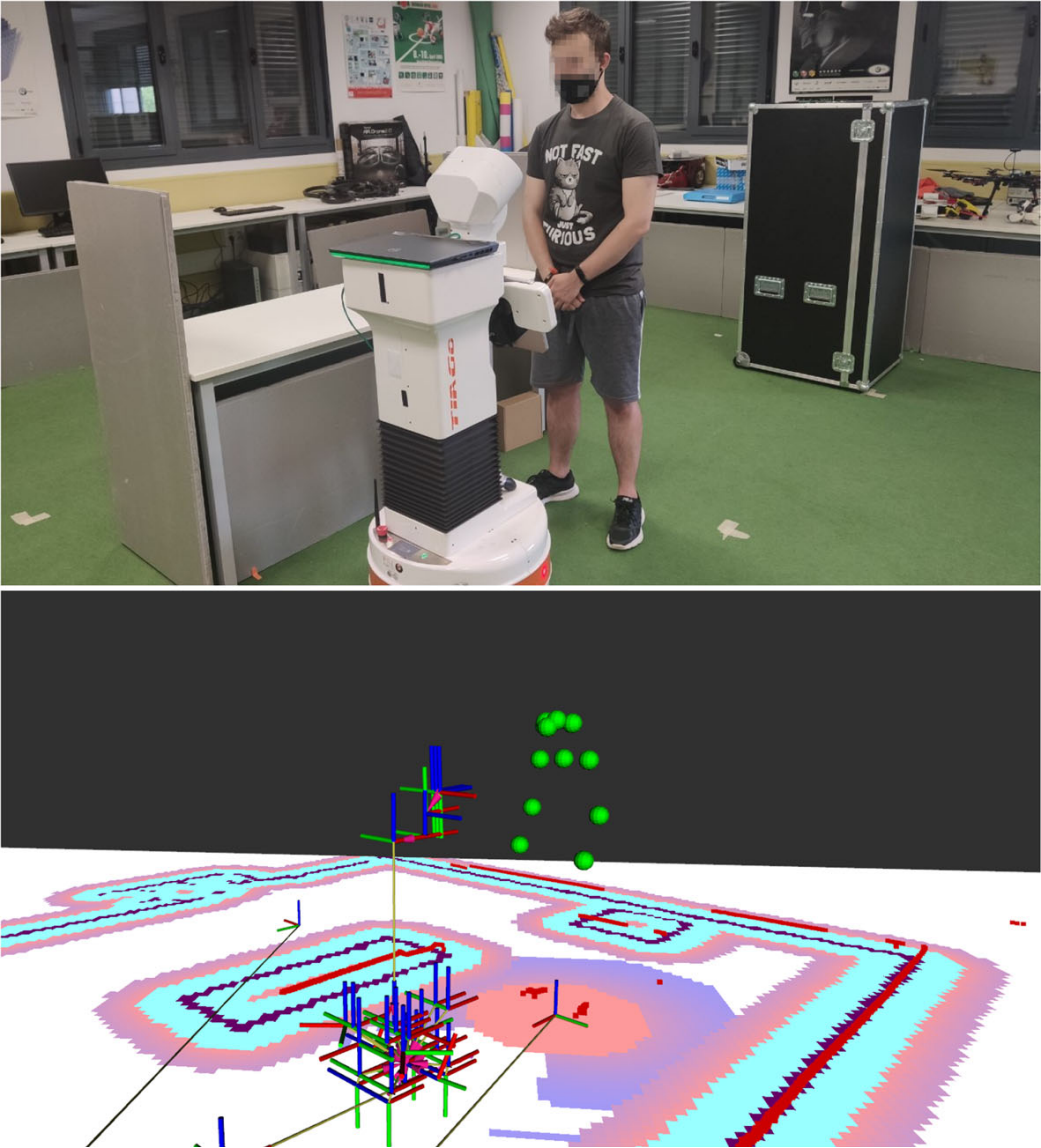
TIAGo robot approaching a person in a controlled scenario

The first scenario compares the performance when it used a mission controller implemented with behavior trees or PDDL actions. In this task, the robot has to approach a human from its rest point and return (Fig. 10). Then, the human’s orientation changes randomly, and the robot has to navigate to the human again. We performed 100 iterations of this experiment with each mission controller. This first scenario was replicate in a real robot (Fig. 11) to confirm that our social_layer and the different decision-making systems are portable, and the full architecture is ready to work in a real application.

**Fig. 12:**
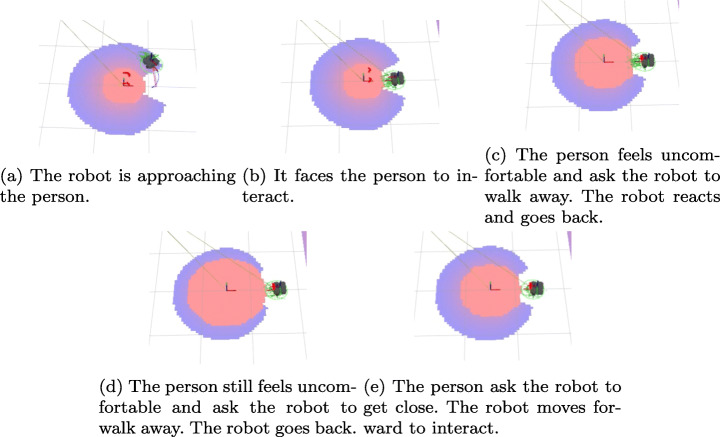
The proxemic zones adapt to the context, e.g. a robot gets too close to a person and he/she, feeling uncomfortable, requests it to walk away. The intimate zone grows to represent the human feelings

The second experiment shows how works the social_layer’s when is integrated into a cognitive architecture. Our social_layer offers the possibility of adapting to human requirements. In this scenario, the robot has to approach a human to talk with him from its rest point. The person requests the robot twice to walk away, and then the individual requests the robot three times to get close. Figure 12 shows how the proxemic zones are modified at runtime based on the person requirements. The information flow between the different components to perform this experiment was presented in Fig. 9. To compare how human comfort is reduced when the robot is asked to move away and does not, we have used metrics already available in the literature.

The navigation speed was set at 0.3 m/s, in the range recommended in [6], the intimate zone radius at 0.45 m, and the personal zone radius at 1.2 m overall the experiments.

### Metrics

It is proposed to analyze the scenarios using the metrics used in [16, 27, 35]. They were formally described in [41]: *d*_*min*_, average minimum distance to a human during navigation; *d*_*t*_, distance traveled; *τ*, navigation time; and *Psi*, personal space intrusions.

These metrics were computed as follows:

- *d*_*min*_: Minimum value in each iteration of the person-robot Euclidean distance in the map.
- *d*_*t*_: Cumulative value of the difference between the robot position in t and t-1 at a frequency of 10 Hz.
- *τ*: Elapsed time between start and iteration end.
- *Psi*: Percentage of the iteration time in which the robot occupies a proxemic zone (personal or intimate). We consider that the robot occupies a zone when any part of the robot base is inside the proxemic zone.

Secondly, this study proposes to measure the system’s general performance when running the different approaches in terms of CPU, memory, and network usage.

Associated with the overall performance, a metric is proposed to evaluate the time to start the navigation process from a stopped position. This value is usually associated with the robot’s acceptability, although we have not focused this study in obtained human feedback.

## Results

The first scenario was repeated in a loop for a hundred iterations. It means that it was running for almost 1 h, where the robot was moving closer and further away in the gazebo simulator.

We take as a baseline the results from our previous work [10], PDDL + DWB. Using PDDL in one loop and Behavior Trees in the other, in combination with DWB and TEB, PDDL + TEB shows a navigation time lower than the time used by the Behavior Trees option, Table 1. On the other hand, the metrics associated with the navigation component present similar behavior, with the low difference in distance traveled and distance to human navigation.

**Table 1:** Comparing a PDDL and a behavior tree decision-making system using different controllers. Social navigation metrics for Approaching Test: for each parameter its mean and standard deviation are provided in parentheses

Parameter	PDDL		Behavior Trees	
	DWB	TEB	DWB	TEB
*τ*(*s*)	89.28 (17.64)	35.09 (6.03)	72.52 (12.65)	41.54 (5.28)
*d*_*t*_(*m*)	7.71 (1.67)	7.97 (1.49)	7.95 (1.28)	8.00 (1.47)
*d*_*min*_(*m*)	0.57 (0.035)	0.63 (0.08)	0.55(0.11)	0.61 (0.08)
*Psi*(*Personal*)(*%*)	7.83 (5.33)	7.97 (7.95)	7.9 (6.33)	7.93 (6.99)
*Psi*(*Intimate*)(*%*)	0 (0)	0 (0)	0.003(0.002)	0 (0)

**Fig. 13:**
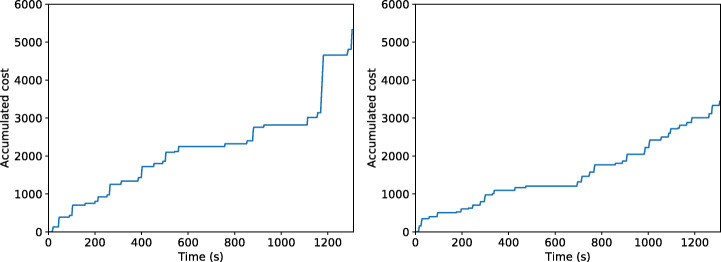
How the robot invades the personal or intimate proxemic zone using DWB (left) and TEB (right)

Focusing only on the controller algorithm, Fig. 13 shows the robot’s proxemic zones invasions over time using DWB or TEB. A lower accumulated cost means higher respect of the human’s proxemic zones by the robot during the experiment, i.e., less impact on the human’s comfort.

In the real-robot experiment, Table 2, the min distance to person was similar to the simulator with the same configuration, but the Psi(Personal) was higher. It was produced because the real scenario is smaller than the simulated, Fig. 11, and the robot had to navigate a little closer to the human to perform its task.

**Table 2:** Metrics for real robot experiment

Parameter	PDDL + TEB
*τ*(*s*)	65.59 (14.59)
*d*_*t*_(*m*)	6.89 (2.17)
*d*_*min*_(*m*)	0.7 (0.12)
*Psi*(*Personal*)(*%*)	12.84 (6.17)
*Psi*(*Intimate*)(*%*)	0 (0)

Finally, Table 3 shows how much time elapsed between the decision-making system starts, and the robot starts moving. The reaction time of the robot is key to satisfy user expectations about robot performance. Delays in the starting time would be understood as errors in robot task execution [34]. Times obtained are quite similar. The difference is imperceptible, notwithstanding the PDDL reaction time is slightly better 0.1 s.

**Table 3:** Time elapsed from the mission controller start up until the robot starts moving

	PDDL	Behavior Trees
Reaction time (s)	0.3008(0.03)	0.4004 (0.0001)

**Fig. 14:**
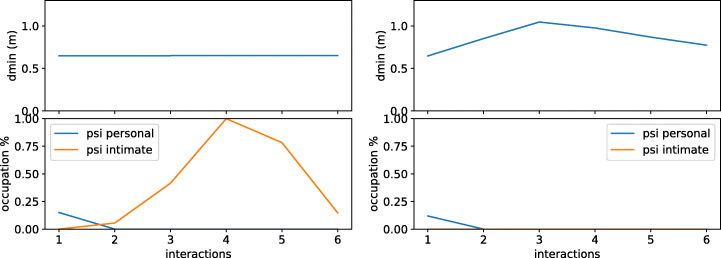
How the robot invades the human’s proxemic zones if it does not adapt to the situation (left) and how the robot reacts to the human requirements (right). Int 1: The robot face the human to interact. Int 2 and 3: The human feels uncomfortable and requests the robot to walk away. Int 4 to 6: The human requests the robot to get close

The second experiment measures the robot’s effects when it does not react to human requirements. Figure 14 shows how the Psi intimate grows when an individual requests a robot to walk away, but the robot does not do anything. Integrating a dialog module with our dynamic proxemic representation framework, we see that the robot can adapt to context to accomplish human needs.

### System performance

**Fig. 15:**
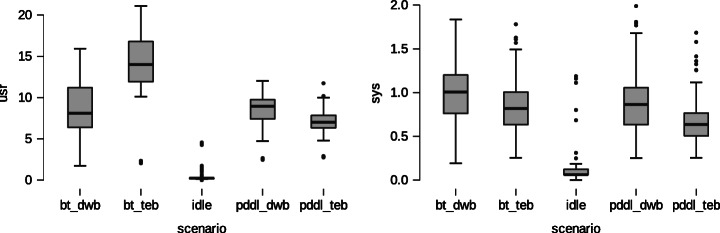
% of CPU consumption by technique

**Fig. 16:**
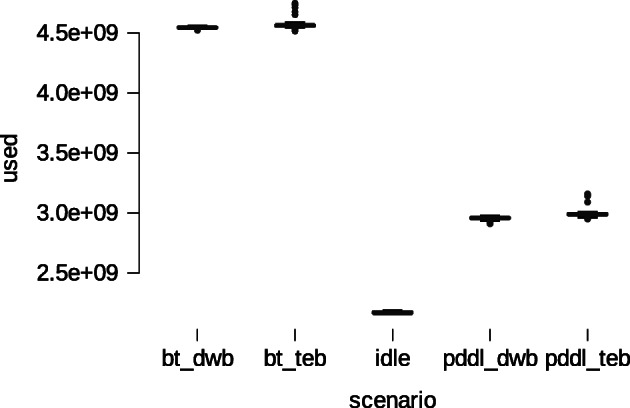
Descriptive statistics of memory consumption

The four techniques described were tested using simulated and real-world experiments. This study tested and analyzed its performance in Gazebo simulator before running it in the robot.For each technique was analyzed its impact on the overall system using *Dstat* command considering three items: 1) CPU stats, aiming to measure CPU usage such as a) user processes and b) system processes; 2) Network stats, aiming to measure the amount of bytes transmitted by the network interface such as a) received and b) sent; 3) memory stats, aiming to measure the amount of memory employed and the total memory available. Figures 15 and 16, present graphically the results using the idle mode as baseline (no techniques running) for comparison.

Figure 15 presents the CPU performance. The Idle mode presents an average of CPU consumption of 0.324(0.534) –Mean(Standard deviation)–. Thus, the values go % of CPU at user level was bt_dwb 8.780 (2.988), bt_teb 14.313(3.280), pddl_dwb 8.498(1.908), pddl_teb 7.163 (1.341). On the other hand the % of system were 0.092 (0.150) by idle, 0.990 (0.310) by bt_dwb, 0.831(0.297) by bt_teb, 0.892 (0.342) by pddl_dwb, and 0.674 (0.247) consumed by pddl_teb. This means, following the user CPU consumption the behavior is quite similar in bt_dwb, pddl_dwb, pddl_teb but slightly higher in bt_teb. Besides, the consumption of sys in four cases is again quite similar. However, TEB behavior is slightly better.

Figure 16 presents the RAM consumption of four techniques. In this case, the performance of PDDL is better than BTS option, having a similar behavior in both DWB/TEB options. Thus, when running in Idle the system spends 2.171e + 9 (7.977e + 6) Bytes, technique bts_dwb shows 4.546e + 9 (5.536e + 6), bts_teb reveals 4.565e + 9 (2.658e + 7), pddl_dwb presents 2.957e + 9 (1.030e + 7) and pddl_teb drains 3.000e + 9 (4.375e + 7) Bytes.

Network consumption under simulation scenario would be simplified, paying attention to outgoing data. The metric in Bytes/second presents values of 8022.533 (3491.013) 8418.506 (3574.225) for bt_dwb, bt_teb respectively and 5317.486 (2375.282), 6622.033 (2572.506) in pddl_dwb and pddl_teb. This means a difference of 37% when comparing pddl_dwb and bt_dwb and 22% when comparing pddl_teb and bt_dwb. However, these results would be omitted when working in simulation.

## Discussion

This study has analyzed two different scenarios to explore the answers to the two research questions proposed. Firstly, this paper analyzes:

- **RQ3**: What are the effects of different behavioral decision-making systems in the activity-aware navigation system?

The first experimental scenario aims to evaluate the effects of each decision-making system in the human-aware navigation system. The results show better time to perform the task in the PDDL approach rather than using behavior trees. This is because social navigation decisions are better modeled when actions are calculated at runtime than when they are preset. If a PDDL model is correct, it optimizes decision-making. In terms of system performance, the IA Planning approach also was better than Behavior Trees.

The second experimental scenario deals with the next research question:

- **RQ4**: What are the effects of different local planner in the activity-aware navigation system?

This study uses two different algorithms for the navigation process: Timed Elastic Band (TEB) and DWB, the updated version of Dynamic Windows Approach algorithm for ROS2. Firstly, the results obtained show a relationship between the navigation algorithm that hides the navigation component Nav2 and robot performance. Our previous experiment in [10] presented a performance of 80 s using DWB version. This study obtained an average of 35 and 41 s using TEB.

Besides, we have analyzed the impact during the task navigation, specifically how long is traversing personal proxemic zone. Findings show that the time spent near the humans is higher in the DWB (Fig. 13) than TEB, so the latter is preferred to perform better in terms of human awareness.

Both scenarios show that it is necessary to have in mind not only the decision-making system for enhancing the human-aware navigation component but also to select the right path-planning algorithm due to its engine to navigate or surround the elements that could be interacting with the robot, such as in this case the distance to the individuals.

**Fig. 17:**
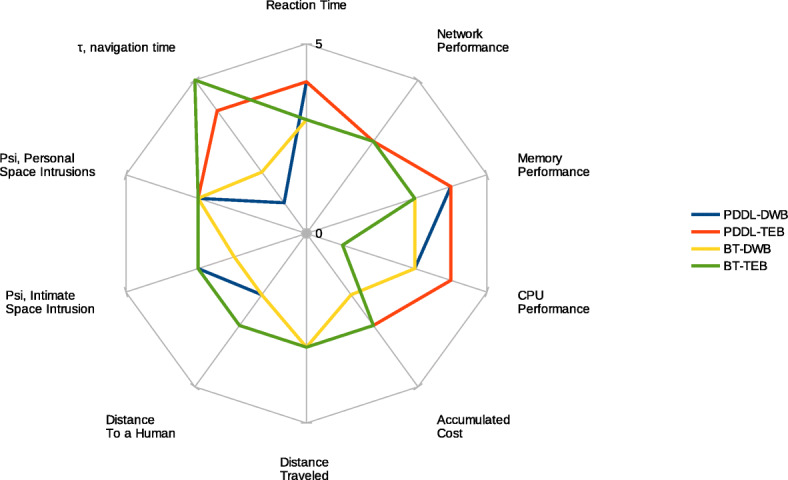
Evaluation of path-planning and decision-making approaches reviewed in this paper 1)Very poor, 2)Poor, 3)Acceptable, 4)Good and 5)Very Good

Finally, this study provides an overview of which is the best option for a human-aware navigation system. Considering the values obtained in the Results section, we have created a table using a scale from 1 to 5. Thus, the quality values are 1)Very poor, 2) Poor, 3) Acceptable, 4) Good, 5) Very Good. Table 4 outlines the values and Fig. 17 presents the values graphically.

**Table 4:** Authors evaluation of decision-making and local planner algorithm examined in this study 1)Very poor, 2)Poor, 3)Acceptable, 4)Good and 5)Very Good

Parameter	PDDL-DWB	PDDL-TEB	BT-DWB	BT-TEB
Reaction Time	4	4	3	3
*τ*, navigation time	1	4	2	5
Psi, Personal Space Intrusions	3	3	3	3
Psi, Intimate Space Intrusion	3	3	2	3
Distance To a Human	2	3	2	3
Distance Traveled	3	3	3	3
Accumulated cost	2	3	2	3
CPU Performance	3	4	3	1
Memory Performance	4	4	3	3
Network Performance	3	3	3	3

In general terms, the combination between PDDL + TEB was the best performer for our application, a social navigation application in which a robot must approach a human to interact. This combination was tested in a real robot with good results. In general terms, the transfer cost between simulator and real-wold was minimum and only increase in one of the metrics, Psi(personal), but keeping in a reasonable range.

## Conclusions

This paper has presented the continuation of our research in social navigation, applying the concept of proxemic. Our research tries to answer how a proxemic framework can be integrated into a cognitive architecture and analyzes the possibilities, implications, and effects. This paper has briefly described our proxemics-based framework for establishing a person’s social zones and how they affect a robot’s navigation in the vicinity and towards a person. We have also described several alternatives to its integration in decision-making systems that are common in cognitive architectures. Finally, we have evaluated each alternative’s impact on a simulated robot interacting with a person.

Of the research questions stated in the introduction to this paper, those related to decision-making systems have been addressed:

- For the question *What are the effects of different behavioral decision-making systems in the activity-aware navigation system?* We conclude that a decision-making system influences the performance of the task and how the task is developed. This effect may be significant in the Human-Robot Interaction task because the human could feel disgusted or frustrated if he/she tries to interact with the robot and the tasks are not executed smoothly.
- For the question *What are the effects of the different local planners in the activity-aware navigation system?* We conclude that choosing the right controller algorithm is decisive for the task’s performance and people’s comfort.

We are extending our research on social navigation in various directions. We have developed a framework for social navigation, and we are taking steps to introduce it into a cognitive architecture. This paper reinforces our opinion that all robot activities should have a social component in people’s presence, with the aim that humans accept a robot and that its existence is more natural for the people with whom it lives. If the cognitive architectures are the ones that direct the behavior of the robot, all its decision-making levels must work in a coordinated way to achieve this goal. Our future work now turns to the upper layers of these cognitive architectures: *What impact does the social component have on a mission planner? Should motivational approaches take into account the social capabilities implemented in a robot?*

## Footnotes

1 http://intelligentroboticslab.gsyc.urjc.es/ros2_planning_system.github.io/

2 https://www.behaviortree.dev/

3 https://github.com/ros-planning/navigation2/blob/main/nav2_dwb_controller/README.md

4 https://github.com/jginesclavero/ros2-tf-pose-estimation
